# Effect of Near-Infrared Pulsed Light on the Human Brain Using Electroencephalography

**DOI:** 10.1155/2021/6693916

**Published:** 2021-03-05

**Authors:** Yi-Chia Shan, Wei Fang, Yang-Chyuan Chang, Wen-Dien Chang, Jih-Huah Wu

**Affiliations:** ^1^Department of Biomechatronics Engineering, National Taiwan University, No. 1, Section 4, Roosevelt Rd., Taipei 10617, Taiwan; ^2^Department of Neurology, Min-Sheng General Hospital, No. 168, Jin-Kuo Rd., Taoyuan, Taoyuan County 330, Taiwan; ^3^Department of Sport Performance, National Taiwan University of Sport, No. 16, Section 1, Shuang-Shin Road, Taichung 404, Taiwan; ^4^Department of Biomedical Engineering, Ming Chuan University, No. 5, Deming Rd., Gweishan Township, Taoyuan 333, Taiwan

## Abstract

In our previous study, the low-level laser (LLL) stimulation at the palm with a stimulation frequency of 10 Hz was able to induce significant brain activation in normal subjects. The electroencephalography (EEG) changes caused by the stimulation of light-emitting diode (LED) in normal subjects have not been investigated. This study aimed at identifying the effects of LED stimulation on the human brain using EEG analysis. Moreover, the dosage has been raised 4 times than that in the previous LLL study. The LED array stimulator (6 pcs LEDs, central wavelength 850 nm, output power 30 mW, and operating frequency 10 Hz) was used as the stimulation source. The LED stimulation was found to induce significant variation in alpha activity in the occipital, parietal, and temporal regions of the brain. Compared to the previous low-level laser study, LED has similar effects on EEG in alpha (8–12 Hz) activity. Theta (4–7 Hz) power significantly increased in the posterior head region of the brain. The effect lasted for at least 15 minutes after stimulation ceased. Conversely, beta (13–35 Hz) intensity in the right parietal area increased significantly, and a biphasic dose response has been observed in this study.

## 1. Introduction

The brainwave rhythm is associated with the physical and mental status. Alpha rhythm is the dominant brainwave in normal adults who are awake and relaxed with their eyes closed, and it decreases under the eyes-open or mentally active condition [1]. The appearance of theta wave is considered to be one of the signs of drowsiness [2]. In individuals with a state of alertness or anxiety, beta activity will increase [3]. Delta waves are the main EEG activity observed during deep sleep. The most dominant pattern of change across several disorder types, including attention deficit hyperactivity disorder (ADHD), schizophrenia, and obsessive-compulsive disorder (OCD), is power increases across lower frequencies (delta and theta) and decreases across higher frequencies (alpha, beta, and gamma) [4]. The alpha waves in the depression group were found to be lower compared to the normal group in both closed eyes and open eyes conditions [5].

Alpha rhythm is an important biomarker to recognize the physiological status. When people feel relaxed and awake in closed eyes, the alpha rhythm is the dominant brainwave [1]. In addition, induced alpha oscillations can be helpful for pain relief. Transcranial alternating current stimulation (tACS) can enhance alpha oscillation in the somatosensory cortex to reduce chronic low back pain [6]. The photic driving response is one of the effective methods to affect brain activity. Photic stimulation with a frequency at or near the native frequency of the posterior dominant rhythm may increase the amplitude of this rhythm. Stimulation with other flash frequencies may cause a photic driving response that replaces or superimposes the posterior dominant rhythm [7]. Stimulation with red stroboscopic light can rapidly and powerfully increase the amplitude of the alpha rhythm in the occipital cortex [8]. In addition, a photic stimulation with LED goggles was applied and found that the alpha rhythms of subjects were highly influenced by different stimulation frequencies [9]. Besides the recognized clinical efficacy of visual stimulation, EEG activity can also be affected by other stimulation modalities, including electric [10], music [11], and magnetic stimulation [12]. In previous studies, a low-level laser was used to stimulate the palm of the subjects under eyes-open and eyes-closed conditions, and such stimulation caused significant changes in the amplitude of the subjects' brainwaves [13, 14].

LEDs radiate noncoherent light, whereas lasers radiate coherent light. Many studies proved LED and laser have similar effects [15–17]. Till now, there are few research studies to discuss the LED stimulation on the skin to induce brainwave activity. The aim of this study is to investigate whether LED light can evoke brainwave activity.

## 2. Methods

The study protocol was approved by the Institutional Ethics Committee of Ming-Sheng General Hospital. Each participant was required to sign an informed consent. This study was performed in conformance with the Helsinki Declaration. The trial was executed at National Taiwan University Hospital.

### 2.1. Participants

Twenty healthy university students (mean age: 21.5 ± 1.3 years, 12 men and 8 women) were recruited for this study. The average physical states of the participants are listed in Table 1. Each subject underwent two trials on different days. They received LED stimulation (the LED group) in one trial and sham LED stimulation (the control group) in the other trial. In the first trial, each subject was randomly assigned to either the LED or the control group. In the second trial, conducted a few days later, each subject was assigned to the group to which they had not been assigned in the previous trial. The exclusive criteria were as follows: (a) a history of psychiatric disorders such as major depression, substance abuse, schizophrenia, or paranoid disorder; (b) cardiopulmonary disease; and (c) medication use currently.

### 2.2. LED Array Arrangement and Dose Calculation

**Figure 1:**
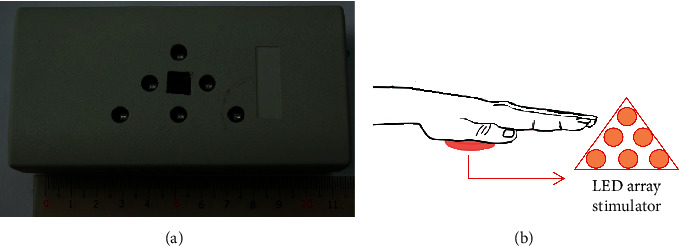
(a) Six LEDs array arranged in a triangle. (b) The schematic of this stimulation.

An array of six LEDs arranged in a triangle, as shown in Figure 1(a), was used to radiate near-infrared (NIR) light onto the palm of subjects. NIR LEDs (model S1VS0850IR002A0Z, Millennium Communication Co., Ltd., Taiwan) were used in this study. The central wavelength, output power, operating frequency, and duty cycle of the LEDs were 850 nm, 30 mW, 10 Hz, and 50%, respectively. The light spot of the LED could be considered to be a circle. The area of the light spot on the skin was approximately 28 mm^2^. Thus, the energy density was calculated to be approximately 32 J/cm^2^ for a 10-minute treatment, the dosage of each LED was 9.0 J, and the total energy was 54.0 J. The irradiation parameters of LED are listed in Table 2. In our previous study, the output power of each laser diode was 7.0 mW, the dosage was 2.1 J, and the total energy was 12.6 J for six laser diodes [13]. The dosage in this study is 4.2 times than that in the laser study. The schematic of this stimulation is shown in Figure 1(b).

### 2.3. Experimental Procedure

Each subject sat in an armchair and placed their left palm on the LED device. The subject was instructed to relax, keep the eyes open, and avoid making any movement. In the LED group, the LEDs were turned on for 10 minutes. In the control group, the LEDs were not turned on. At the beginning of each trial, each subject relaxed for 5 minutes to achieve a stable physiological state. Ongoing EEG activity was recorded under the eyes-open condition at three stages (6 sessions): before stimulation (baseline 5 minutes, session 1), during stimulation (LED stimulation, 10 minutes, sessions 2 and 3), and after stimulation (post-LED stimulation, 15 minutes, and sessions 4, 5, and 6). This procedure was the same as that followed in our previous study [13, 14].

### 2.4. EEG Recording and Measurement

**Figure 2:**
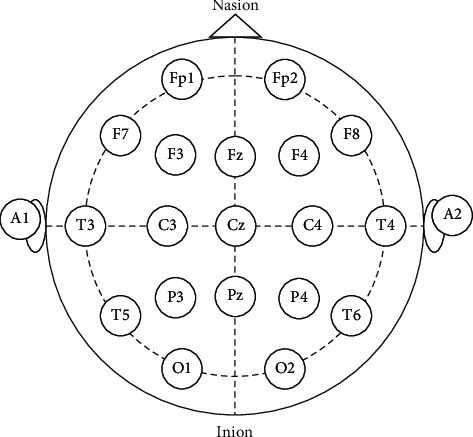
10–20 system of EEG electrode placement.

**Figure 3:**
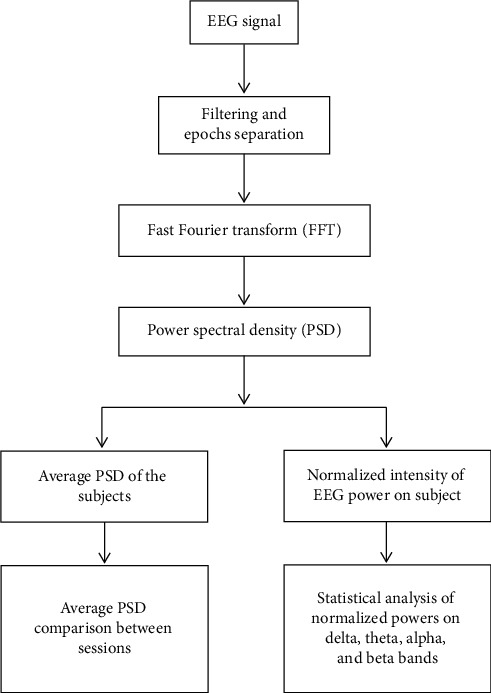
The flowchart of the EEG signal analysis.

An EEG instrument (Neurofax model EEG-1000, NIHON KOHDEN) was used in this study. According to the international 10–20 system as shown in Figure 2, Ag/AgCl electrodes placed on the scalp were used to record the variation of cerebral activity. A bipolar recording technique was used to record the potential differences at Fp2-F4, F4-C4, C4-P4, P4-O2, Fp1-F3, F3-C3, C3-P3, P3-O1, Fp2-F8, F8-T4, T4-T6, Fp1-F7, F7-T3, and T3-T5. Each set of EEG data (5 minutes epoch) was transformed to the EEG band (*μv*^2^) with fast Fourier transform (FFT) in four frequency bands: delta (0.5–3.5 Hz), theta (4–7 Hz), alpha (8–13 Hz), and beta (13–50 Hz). For each session, the four band powers of valid epochs were averaged, and FFT maps of different sessions were constructed using Neurofax version 05–80. The flowchart of the EEG signal analysis is shown in Figure 3.

### 2.5. Statistical Analysis

A two-tailed paired *t*-test was employed to compare the difference in the EEG band power before and after the actual or sham LED stimulation. The power recorded at Fp2-F4, F4-C4, C4-P4, P4-O2, Fp1-F3, F3-C3, C3-P3, P3-O1, Fp2-F8, F8-T4, T4-T6, Fp1-F7, F7-T3, and T3-T5 was investigated. The mean and standard deviation of the calculated values were expressed as mean ± SD. All statistical analyses were performed using SPSS software, and the results with a *p* value <0.05 were considered statistically significant.

## 3. Results

### 3.1. Alpha Activity and Latent Effect

**Figure 4:**
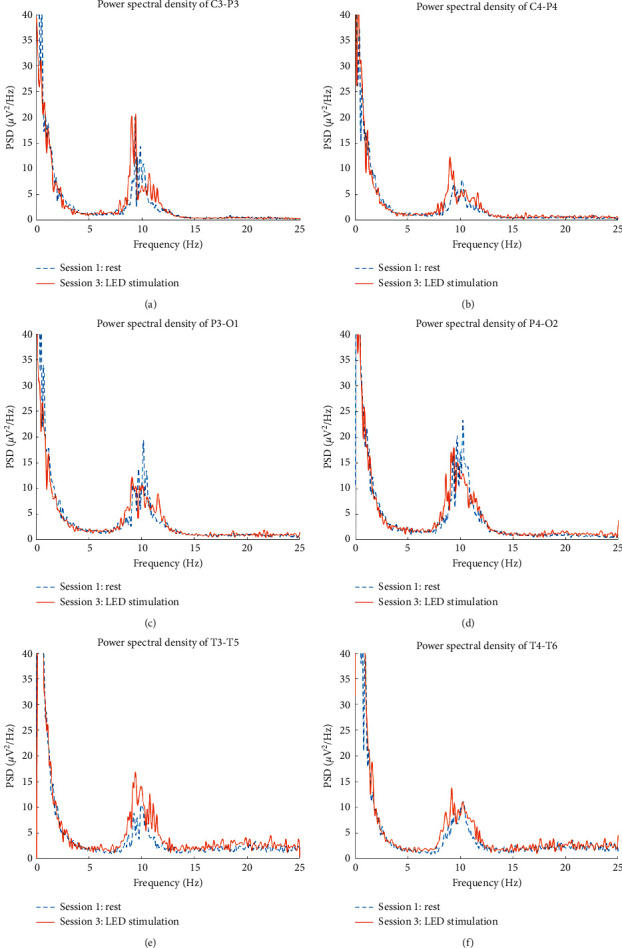
The average power spectral density of the subjects in the parietal, occipital, and temporal cortex locations during session 1 (rest) and session 3 (LED stimulation). (a) C3-P3 location during session 3 stimulation and session 1 rest. (b) C4-P4 location during session 3 stimulation and session 1 rest. (c) P3-O1 location during session 3 stimulation and session 1 rest. (d) P4-O2 location during session 3 stimulation and session 1 rest. (e) T3-T5 location during session 3 stimulation and session 1 rest. (f) T4-T6 location during session 3 stimulation and session 1 rest.

**Figure 5:**
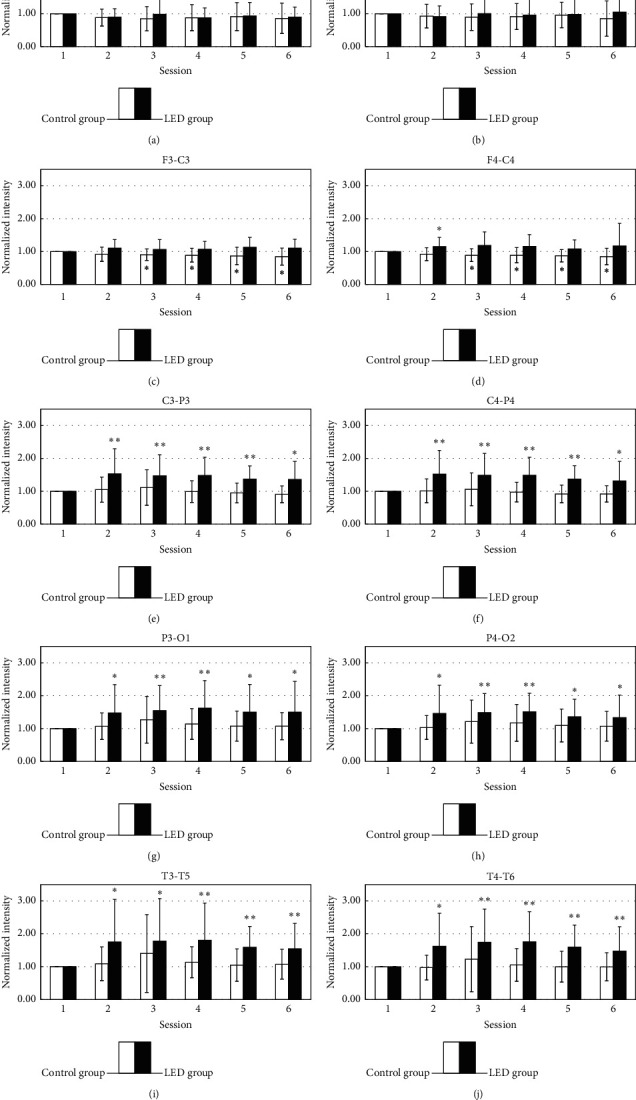
The statistical analysis of the alpha band (8–13 Hz) by comparing the baseline and each session in LED and placebo group is shown in different areas. ^*∗*^*p* < 0.05 by paired-sample *t*-test. ^*∗∗*^*p* < 0.01 by paired-sample *t*-test.

There are six sessions in the study. The alpha power of session 1 (rest) and session 3 (LED stimulation) was compared because session 3 is the maximum variation in six sessions. The average power spectral density (PSD) of the subjects in parietal, occipital, and temporal cortex locations during session 1 and session 3 is presented in Figure 4. The PSD in the alpha band was shifted toward low frequencies after stimulation. In addition, the total PSDs in the alpha band also have been enlarged. In order to reduce the influence of some objects, a normalized power calculation had been used. The average power calculated for each session was normalized by dividing it by the corresponding data in the first session.  Figure 5 illustrates the temporal changes in the normalized intensity of EEG power in the alpha band. In the LED group, alpha power significantly increased from session 2 to session 6, in these areas (C3-P3, C4-P4, P3-O1, P4-O2, T3-T5, and T4-T6). A similar effect was observed on both sides of the posterior head region. During session 2 to session 3, the intensity was increased by LED irradiation. In addition, the intensity after LED irradiation was maintained from session 4 to session 6, although there was a slight decrease. The latent effect was maintained for at least 15 minutes after LED ceased. In the control group, there was no variation in the alpha power in the posterior head region.

### 3.2. Other Brainwave Activities

**Figure 6:**
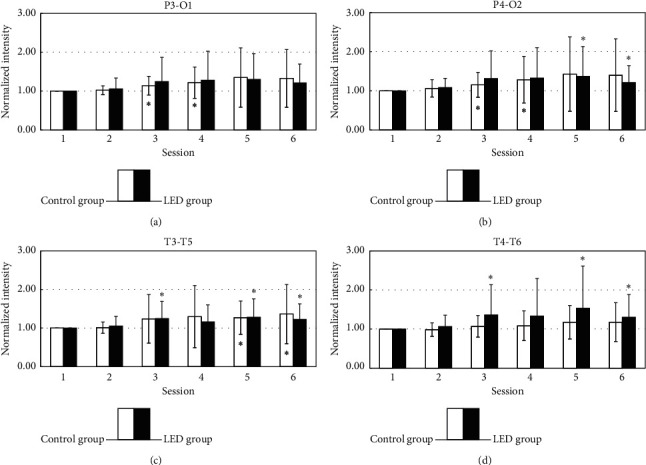
The statistical analysis of the theta band (4–7 Hz) by comparing the baseline and each session in LED and placebo group is shown in different areas. ^*∗*^*p* < 0.05 by paired-sample *t*-test. ^*∗∗*^*p* < 0.01 by paired-sample *t*-test.

Figure 6 displays the temporal changes in the normalized intensity of EEG power in the theta band. In both groups, theta power increased from session 2 to session 6 in the posterior head region (P4-O2, T3-T5, and T4-T6). In the right temporal area (T4-T6), the intensity of the theta band in the LED group was higher than that in the control group in sessions 3 to 6.

**Figure 7:**
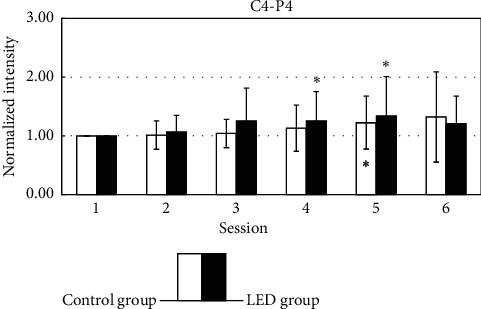
The statistical analysis of the beta band (13–50 Hz) by comparing the baseline and each session in LED and placebo group is shown in different areas. ^*∗*^*p* < 0.05 by paired-sample *t*-test. ^*∗∗*^*p* < 0.01 by paired-sample *t*-test.

The temporal changes of the normalized intensity of EEG power in the beta band are shown in Figure 7. Among them, beta intensity in the right parietal area (C4-P4) increased significantly in sessions 4 to 5 of the LED group.

Most of the EEG power in the delta band has no significant meaning, so the results were omitted for a concise reason.

The significant variations in the normalized power in the brain region with LED stimulation are listed in Table 3, and the affected regions with laser stimulation [13] were also included.

## 4. Discussion

### 4.1. Brainwave Activity at EEG Frequency Band in LED Stimulation

Alpha rhythm is the dominant brainwave in normal adults who are awake and relaxed with their eyes closed. Alpha rhythm is primarily located in the posterior head region, and it decreases under the eyes-open or mentally active condition [1]. Audiovisual stimulation (AVS) is a familiar and useful method to affect brainwave activity. Brainwave entrainment was harmonic with the visual stimulation frequency, but the latent effect is short, no consistent pattern of persistent excited power was found in few minutes after AVS [18]. In this experiment, the average PSD of the subjects in the parietal and temporal cortex locations during session 3 was increased and shifted toward lower frequencies in comparison with the rest stage in Figure 4. The phenomenon was caused by LED stimulation which was operated at 10 Hz. The normalized alpha activity rapidly increased during and after LED stimulation in the LED group in Figure 5. The affected regions were the occipital, parietal, and temporal regions. LED stimulation exerted a latent effect on alpha rhythm. The effect could persist for at least 15 minutes after LED ceased. Based on the experimental results, LED stimulation at the palm has an effect to induce alpha rhythm activity, and the affected regions were distributed in posterior. The influence was like AVS, but the latent time was longer. In the control group, no significant variation in alpha rhythm was observed.

Theta waves in the range are generally regarded as abnormal in awake adults. The emergence of theta waves is considered one of the hallmarks of the onset of drowsiness [2]. Moreover, theta activity is associated with attention and the efficient processing of cognitive and perceptual tasks [3, 19]. Lagopoulos et al. found that both alpha and theta activities increased during nondirectional meditation under the eyes-closed condition. Theta activity was significantly higher in the frontal and temporal-central regions than in the posterior region [20]. In the present study, theta activity was increased in the posterior head region in the LED group. In particular, the affected region was in the right temporal area (T4-T6), and its intensity was increased significantly. It could be related to the contralateral effect, LED stimulated at the left palm, and theta wave activated in the right hemisphere brain.

Beta activity increases in individuals who are alert [3], anxious, or have their eyes open. Beta activity is usually predominant in the frontal and central regions. In the present study, slight variations and no significant LED-induced changes were observed in beta power, except in C4-P4 position, and we will discuss it later.

Delta waves are not observed in normal awake adults, but they are the main EEG activity observed during deep sleep. Blinking or eyeball movement usually causes artifacts, which resemble EEG waves in the delta range. In this study, since there was no significant variation in delta power, the data were omitted.

### 4.2. Brainwave Activity in Coherence Light vs. Noncoherence Light

Several studies have discussed the differences in the biological effects exerted by coherent and noncoherent light irradiation. Rochkind et al. reported that low-level laser (LLL) therapy (632.8 nm, 15 mW, 10 J/cm^2^) exerted a short-term effect on an injured peripheral nerve, but noncoherent light (660 nm, 10 mW) exerted a weak less effect [21]. Stasinopoulos et al. used Ga-As LLL therapy (904 nm, 3.51 J/cm^2^; a total of six points) and polarized polychromatic noncoherent light (Bioptron light: 480–3400 nm, 2.4 J/cm^2^) in combination with an exercise program to treat lateral elbow tendinopathy [22]. They found no significant differences in the reduction of pain and the improvement of function between the Ga-As LLL therapy and the Bioptron light groups. Bertoloni et al. discovered similar biochemical and morphological changes in *Escherichia coli* after irradiation with coherent or noncoherent light [23]. Demidova-Rice et al. compared the effects of coherent light (He-Ne laser) and noncoherent light on excision wound healing in mice and identified no significant difference in the mean healing curve obtained using the light sources [16]. Agnol et al. reported that noncoherent LED light (640 nm with 40 nm full bandwidth at half maximum) exerted similar or even better biostimulus effects than coherent laser light (660 nm, 6 J/cm^2^) [17]. For tissue repair in diabetic rats, they observed that treatment with LEDs was more efficient in reducing wound size than treatment with a laser. Debates about any differences in the effects of coherent and noncoherent light irradiation can be found in the literature. However, different experimental parameters might be responsible for the differences in biological effects exerted by these two light source types. For example, the irradiation dosages of noncoherent light were not mentioned in some studies [17, 21], and the range of wavelength of polychromatic light was wide from visible to infrared (480–3400 nm) [22]. In literature studies from Karu [24], the main influence parameters of the low-power light stimulation on the physiological state are the irradiation wavelength, power, dose, the operating frequency, and the time duration, while coherent and polarized light have no significant effect. In this study, we found alpha rhythm increase obviously and significantly in the posterior region, the phenomenon is like the laser stimulation [13]. It means that the noncoherent light and coherent light have a similar biological effect under similar parameters setting.

### 4.3. Dosage and Conduction Pathway

**Figure 8:**
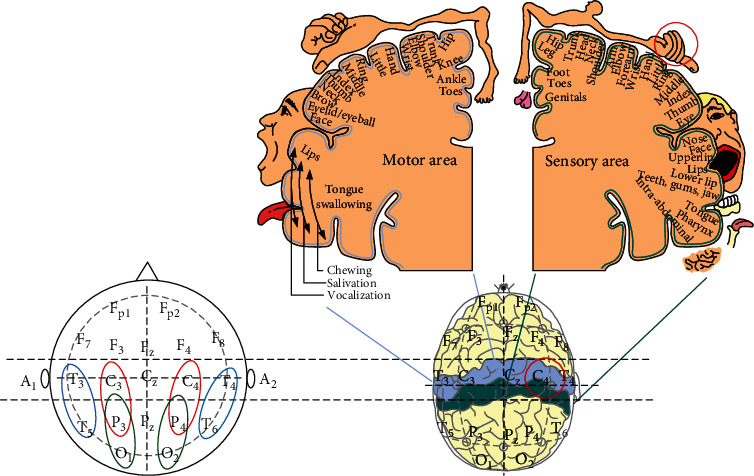
Alpha power significantly increased in the occipital, parietal, and temporal regions, as shown in the left corner small figure. The activation C4-P4 reflected the palm's higher stimulation as shown in the right corner small figure.

Regarding LED- and laser-induced EEG changes [13], both light sources exert similar effects on EEG power in the alpha band of brainwaves. It is worthy to mention the normalized intensity of alpha activity during and after LED stimulation was 1.50–1.55 times higher than the initial intensity at C3-P3 and C4-P4 in sessions 2 and 3; however, in laser study [13], the increment is 1.20–1.35. Comparing LED and laser stimulation, the normalized intensity of alpha activity induced by LED irradiation was higher than that induced by laser irradiation. This difference may be attributed to the irradiation dosage; the LED dosage (54.0 J) was higher than the laser dosage (12.6 J) [13]. Alpha power significantly increased in the occipital, parietal, and temporal regions, as shown in the left corner small figure in Figure 8. Besides, the beta power in C4-P4 position was raised slowly in session 2 (stimulation session) and raised in session 4 and session 5, and it has a significant meaning. However, in our previous laser study [13], the beta power in C4-P4 position did not change. It means the more powerful the stimulation, the more the nerve response. The left palm of the subjects was stimulated with higher stimulation, the activation C4–P4 reflected the palm's stimulation as shown in the right corner small figure in Figure 8 [25, 26], and even sensorimotor cortex activation can be observed in infancy [26]. According to the LED and laser test results, the higher the dose, the higher the alpha and theta activities, and there is a positive correlation between the dose and the alpha and theta activities. On the contrary, higher LED dosage induced the beta activity of the right parietal hemisphere, but lower laser dosage reduced the beta activity [13]. The response seems to follow the “Arndt–Schulz” law and a biphasic dose response has been observed [27]. It means that insufficient energy cannot promote biological effects. If more energy were applied across the threshold, the response will be achieved. But too much energy results in biological inhibition.

### 4.4. Effect in Pulse Light

The pulse frequency is also a factor to cause the biological effect. The calcium uptake in macrophages is increased after pulsed diode laser irradiation [28]. The chemiluminescence of the mouse is enhanced after being irradiated with a pulsed laser [29]. The difference of pulsed laser versus electrical energy for peripheral nerve stimulation in the animal model has been investigated [30], and they proved that the use of a pulsed laser exhibits distinct advantages when compared to standard electrical means for excitation of muscle potentials in the peripheral nerve. Mathew et al. found that continuous wave (CW) light does not produce any significant influence on the axon growth. In contrast, when using pulsed light, the beam was able to modify the trajectory of the axons, attracting approximately 45% of the observed cases to the beam spot [31]. Comparing the effects of pulsed and CW in low-level light therapy, there is evidence that pulsed light does have a different effect than CW light and pulsed light has a better effect [32].

In this study, LED stimulation induced significant activation of alpha rhythms in normal subjects. This finding implies that LED stimulation operated at different frequencies similar to laser stimulation may have the potential to induce specific brainwave in individuals. Thus, LED stimulation with the appropriate LED power and operating frequency may enable a person to relax or fall asleep easily.

## 5. Conclusion

This study applied LED stimulation to normal subjects, and stimulation-induced changes in EEG power were analyzed. Alpha power significantly increased in the occipital, parietal, and temporal regions. Theta power significantly increased in the posterior head region. The enhancements were maintained for at least 15 minutes. We found that LEDs and lasers exert similar effects on alpha rhythm. The effect is dependent on the stimulation dosage, and a biphasic dose response has been observed in these studies. The higher the dosage, the higher the alpha activity in both hemispheres. Conversely, higher LED dosage (54.0 J) induced the beta activity of the right parietal hemisphere, but lower laser dosage (12.6 J) reduced beta activity. The major limitation of the present study refers to the subject recruited in this study. The present study is not informative with respect to the severity or timeframe of sleep disturbances. Sleep quality is an important factor that affects the outcome of brainwave after light stimulation. Future studies might try to capture other more detailed and more objective indicators of sleep quality. Furthermore, the therapeutic window for inducing specific brainwaves via light stimulation has been found. We believe that the results in this study have practical implications in the medical field. For instance, using a higher dose (or frequency) of light stimulation may have the potential application in enhancing the attention of students. Conversely, using a low dose of light stimulation may improve the sleep problems for people who have insomnia. It is worthy to further study.

## Figures and Tables

**Table 1:** Average physical status of the participants.

Physical status	Average value
Age (years)	21.50 ± 1.30
Weight (kg)	60.25 ± 7.69
Height (cm)	162.60 ± 7.04
BMI (kg/m^2^)	22.75 ± 2.17

**Table 2:** The irradiation parameters of LED.

Parameters	Value
LED wavelength	850 nm
Pulse frequency	10 Hz
Duty ratio	50%
Peak power/pc	30 mW
Treatment time	10 min
Energy/pc	9 J
Irradiation spot area/pc	28 mm^2^
Energy density/pc	32 J/cm^2^
Total energy (6 pcs)	54 J

**Table 3:** Significant variation in the normalized power in the brain region with stimulator.

Stimulator		LED (30 mW)	Laser (7 mW) [13]
Brainwave	Beta	C4-P4 increase **↑**	T3-T5, T4-T6, F7-T3, F8-T4 decrease **↓**
	Alpha	C3-P3, C4-P4, P3-O1, P4-O2, T3-T5, T4-T6 increase **↑↑**	C3-P3, C4-P4, P3-O1, P4-O2, T3-T5, T4-T6 increase **↑↑**
	Theta	P4-O2, T4-T6, T3-T5 increase **↑**	P3-O1, P4-O2, T4-T6 increase **↑**
	Delta	None variation	None variation

## Data Availability

The data used to support the findings of this study are included within the article.
